# *Dermatophagoides farinae* microRNAs released to external environments via exosomes regulate inflammation-related gene expression in human bronchial epithelial cells

**DOI:** 10.3389/fimmu.2023.1303265

**Published:** 2023-12-01

**Authors:** Kaiyue He, Ting Yang, Jinyan Yu, Xiao Zang, Shangde Jiang, Shuyue Xu, Jiaxi Liu, Zuyu Xu, Wei Wang, Shanchao Hong

**Affiliations:** ^1^ Department of Clinical Laboratory, Jiangnan University Medical Center, Wuxi, Jiangsu, China; ^2^ Department of Dermatology, Affiliated Children’s Hospital of Jiangnan University, Wuxi, Jiangsu, China; ^3^ Graduate School of Nanjing Medical University, Nanjing, Jiangsu, China; ^4^ School of Public Health, Nanjing Medical University, Nanjing, Jiangsu, China; ^5^ National Health Commission Key Laboratory on Parasitic Disease Prevention and Control, Jiangsu Provincial Key Laboratory on Parasites and Vector Control Technology, Jiangsu Institute of Parasitic Diseases, Wuxi, Jiangsu, China

**Keywords:** *Dermatophagoides farinae*, miRNAs, exosomes, living environment, cross-species regulation, inflammation-related processes

## Abstract

**Background:**

*Dermatophagoides farinae* (DFA) is an important species of house dust mites (HDMs) that causes allergic diseases. Previous studies have focused on allergens with protein components to explain the allergic effect of HDMs; however, there is little knowledge on the role of microRNAs (miRNAs) in the allergic effect of HDMs. This study aimed to unravel the new mechanism of dust mite sensitization from the perspective of cross-species transport of extracellular vesicles-encapsulated miRNAs from HDMs.

**Methods:**

Small RNA (sRNA) sequencing was performed to detect miRNAs expression profiles from DFA, DFA-derived exosomes and DFA culture supernatants. A quantitative fluorescent real-time PCR (qPCR) assay was used to detect miRNAs expression in dust specimens. BEAS-2B cells endocytosed exosomes were modeled *in vitro* to detect miRNAs from DFA and the expression of related inflammatory factors. Representative dfa-miR-276-3p and dfa-novel-miR2 were transfected into BEAS-2B cells, and then differentially expressed genes (DEGs) were analyzed by RNA sequencing. Protein-protein interaction (PPI) network analysis and Kyoto Encyclopedia of Genes and Genomes (KEGG) pathway and Gene Ontology (GO) terms enrichment analyses were performed on the first 300 nodes of DEGs.

**Results:**

sRNA sequencing identified 42 conserved miRNAs and 66 novel miRNAs in DFA, DFA-derived exosomes, and DFA culture supernatants. A homology analysis was performed on the top 18 conserved miRNAs with high expression levels. The presence of dust mites and miRNAs from HDMs in living environment were also validated. Following uptake of DFA-derived exosomes by BEAS-2B cells, exosomes transported miRNAs from DFA to target cells and produced pro-inflammatory effects in corresponding cells. RNA sequencing identified DEGs in dfa-miR-276-3p and dfa-novel-miR2 transfected BEAS-2B cells. GO and KEGG enrichment analyses revealed the role of exosomes with cross-species transporting of DFA miRNAs in inflammatory signaling pathways, such as JAK-STAT signaling pathway, PI3K/AKT signaling pathway and IL-6-mediated signaling pathway.

**Conclusion:**

Our findings demonstrate the miRNAs expression profiles in DFA for the first time. The DFA miRNAs are delivered into living environments via exosomes, and engulfed by human bronchial epithelial cells, and cross-species regulation may contribute to inflammation-related processes.

## Introduction

House dust mites (HDMs) are one of the most important and most common indoor allergen sources worldwide that lead to asthma and other respiratory allergic diseases (1), and *Dermatophagoides farinae* (DFA) and *D. pteronyssinus* (DPT), two common species of HDMs, are widely prevalent in temperate and tropical regions (2, 3). It is estimated that 65 to 130 million people worldwide are affected by HDMs allergy (3). Among 129 patients diagnosed with HDM-induced allergic rhinitis (AR) alone for the first time in Department of Allergy, Beijing Tongren Hospital, China during the period from December 2019 to April 2021, the detection rates of Der f 1 and Der f 2, which were associated with moderate AR, were 68.2% and 76.64% (4).

In daily living environments, HDMs release many allergens, including mite bodies, excretions and secretions, and the main particles containing HDMs allergens are fecal particles with 20 to 25 μm in diameter (5). Most individuals with respiratory allergies present IgE responses to Der f 1 and Der f 2, which show sero-dominant specificities (6). However, these DFA allergens fail to explain the exact sensitization mechanism of DFA. MicroRNAs (miRNAs), a group of non-coding small RNAs (sRNAs) composed of 19 to 22 nucleotides in length, are involved in post-transcriptional regulation of multiple biological processes (7). Accumulating evidences have shown that miRNAs play a critical regulatory role in allergic airway diseases (8). MiRNA let-7 was reported to present proinflammatory effects in experimental asthma (9), and overexpression of miR-1 reduces asthma phenotype in endothelial cells and alleviates airway eosinophilia in mouse models (10). Our previous study presented the successful isolation of DFA-derived exosomes and profiled the expression of miRNAs from DFA-derived exosomes using sRNA sequencing (sRNA-Seq) (11). However, there is no knowledge on the expression profile of DFA miRNAs, and the functions of DFA miRNAs remain to be unraveled in allergy.

In this study, we profiled miRNAs expression in DFA mites, mimicked daily living environments in laboratory using a solid culture medium for DFA and sequenced miRNAs expression profiles in the solid culture supernatants of DFA. Next, PCR assay confirmed the presence of both DFA and DPT mites in all three living environments, including sofas, carpets, and mattresses, while quantitative fluorescent real-time PCR (qPCR) assay was used to detect miRNAs in dust mites. We confirmed the secretion of DFA-derived exosomes into external environments and found internalization of DFA-derived exosomes by bronchial epithelial BEAS-2B cells and high expression of DFA-specific miRNAs in BEAS-2B cells. In addition, internalization of these exosomes promoted the release of inflammation-related factors, and two highly abundant DFA-specific miRNAs dfa-miR-276-3p and dfa-miR-novel2 were transfected into BEAS-2B cells to investigate the biological functions of DFA miRNAs in bronchial epithelial cells. Our study may provide insights into the mechanisms underlying of DFA-induced allergy.

## Materials and methods

### DFA mites sampling

DFA mites were cultured in a culture medium consisting of yeast, starch, and rice flour at 25°C in a small air-filtered room with 70% relative humidity. An acupuncture needle was used to pick DFA mites from the culture medium and mite samples were transferred to 1.5 mL EP tubes containing TRIzol Reagent (TaKaRa; Shiga, Japan). A total of 200 dust mites were collected using this approach and stored at −80 °C for sequencing by BGI (Beijing, China). We isolated DFA-derived exosomes using the method described in our previous report (11). To obtain DFA culture supernatants, the solid culture medium of DFA was dissolved with sterile 1 × phosphate buffered saline (PBS; Gibco, Grand Island, NY, USA). The mixture was stirred, precipitated for 2 hours, and the obtained supernatant was filtered through a 40 μm cell strainer (Corning, Inc.; Corning, NY, USA). The resultant supernatant was centrifuged at 3,500 × *g* for 40 minutes, followed by centrifugation at 10,000 × *g* for 1 hour at 4°C. The supernatant was filtered through a 0.22 μm cell strainer (Corning, Inc.) and the obtained supernatant was stored at −80°C for subsequent experiments.

### sRNA-Seq analysis

Total RNA was isolated from DFA mites, DFA-derived exosomes and DFA culture supernatants using the TRIzol Reagent, and RNA quantity and quality were measured with Agilent DNF-471 RNA Kit (15 nt) and Agilent 2100 Bioanalyzer (Agilent Technologies, Inc.; Santa Clara, CA, USA). About 500 ng total RNA was isolated on a denaturing polyacrylamide gel, and sRNAs ranging from 18 to 30 nt in length were excised and ligated sequentially from 5′ to 3′ ends. Subsequently, adaptor primers were added to amplify RNAs, and RNA was purified from denaturing polyacrylamide gels. Following library construction, sRNA was sequenced with the BGISEQ-500 High-throughput Sequencing Platform (BGI; Shenzhen, China). Stringent quality control was performed at each sequencing procedure to ensure the reliability of sequencing data, and detailed sequencing procedures were described in our previous study (11). Following removal of low-quality tags, there were 46,095,191, 40,126,626 and 40,134,247 clean tags yielded from DFA mites, DFA-derived exosomes and DFA culture supernatants, respectively, and the length distribution of sRNA is displayed in **Supplementary Figure S1**. All raw sequencing data generated from this study have been deposited in NCBI BioProject (accession number: PRJNA1023682). Clean tags were then filtered and mapped to the miRBase database (http://www.mirbase.org/). Based on exact matching of identified miRNAs to any known miRNAs in the miRBase database, the yielded miRNAs were categorized into two groups: conserved and novel miRNAs. If a DFA miRNA shared at least one identity with a known miRNA in the miRBase database, it was considered as a conserved miRNA, and the remaining specific miRNAs were defined as novel miRNAs.

### Dust collection and analysis

Dust samples from sofas, mattresses, curtains and carpets of inhabited houses, vacant houses and student dormitories were collected. Then, 5 g dust samples were suspended and stirred with 40 mL PBS in 50 mL centrifugation tubes. Nested PCR assay was performed to identify DFA and DPT in dust samples with specific primers as previously described (12, 13). The mixture was placed for 4 hours at 4°C, and then the obtained supernatant was centrifuged at 3,500 × *g* for 40 minutes, followed by centrifugation at 10,000 × *g* for 1 hour at 4°C. The supernatant was filtered through a 0.22 μm cell strainer (Corning, Inc.) and used for qPCR analysis of miRNAs.

### Cell line and culture

Human bronchial epithelial BEAS-2B cells were purchased from Procell Biological Company (Shanghai, China), and cultured on plastic aseptic dishes in Dulbecco’s modified Eagle’s medium (DMEM; Thermo Fisher Scientific, Waltham, MA, USA) supplemented with 10% fetal bovine serum (FBS; Sigma-Aldrich, Darmstadt, Germany) and 1% penicillin (100 IU/mL; Sigma-Aldrich, Darmstadt, Germany)-streptomycin (100 mg/mL; Sigma-Aldrich, Darmstadt, Germany). Cells were maintained at a humidified atmosphere containing 5% CO_2_ at 37°C.

### Characterization, labeling and tracking of DFA-derived exosomes

DFA culture supernatants was centrifuged at 120,000 × *g* for 2 hours at 4°C, and the bright yellow gel-like precipitate was further washed with sterile PBS, with additional ultracentrifugation required under the same conditions. The bright yellow gel-like pellet was then re-suspended in sterile PBS. The morphology of DFA-derived exosomes was identified using a JEM-1400 transmission electron microscope (JEOL; Tokyo, Japan), and the particle sizes of DFA-derived exosomes were measured using the ZetaView^®^ PMX110 nanoparticle tracking analysis (NTA) instrument (Particle Metrix; Meerbusch, Germany). Three video cycles were recorded and data were analyzed with the software ZetaView 8.04.02 SP2 (Particle Metrix).

DFA-derived exosomes were labeled using the cell membrane staining kit PKH26 (BestBio; Shanghai, China) following the manufacturer’s instructions. In brief, PKH26 dye was mixed with DFA-derived exosomes and incubated in the darkness for 20 minutes. The mixture was transferred to a 100 kD ultrafiltration tube (Millipore; Bedford, MA, USA) containing 10 mL PBS. The isolated PKH26-labelled exosomes were added to BEAS-2B cells following centrifugation at 1,000 × *g* for 30 minutes. Following 24-hour incubation, the cellular uptake of PKH26-labelled exosomes by BEAS-2B cells was observed using immunofluorescence staining. PBS + 2% bovine serum albumin (BSA; Thermo Fisher Scientific) was used to block non-specific binding sites for 30 min at 4°C. DFA-derived exosomes were stained with PKH26 dyes. All exosome samples were visualized with an IX71 fluorescence microscope (Olympus; Tokyo, Japan).

### qPCR assay

Total RNA was extracted from DFA mites and BEAS-2B cells using the TRLzol Reagent (TaKaRa; Beijing, China), and total RNA was extracted from DFA-derived exosomes and DFA culture supernatants using the EasyPure^®^ RNA Kit (TransGen Biotech; Beijing, China). RNA quantity was measured with the NanoDrop Ultramicro spectrophotometer (Thermo Fisher Scientific). MiRNAs and mRNAs were reversely transcribed into cDNA using the riboSCRIPT Reverse Transcription Kit (RiboBio; Guangzhou, China) and the PrimeScript RT Master Mix kit (TaKaRa), and miRNAs and mRNAs expression was quantified with the miDETECT A Track™ miRNA qRT-PCRStarter Kit (RiboBio) and the PerfectStart^®^ Green qPCR SuperMix Kit (TransGen Biotech) using the designed specific primers (**Table 1**) on the Applied Biosystems^®^ 7500 Real-time PCR Systems (Thermo Fisher Scientific). Relative expression of miRNAs and mRNAs normalized to *cel-39* and *β-actin* was calculated using the 2^-ΔΔCt^ method. In addition, the expression of total miRNAs was checked using electrophoresis on 1.5% agarose gels, and the results were visualized with the gel imaging system (Bio-Rad; Hercules, CA, USA).

**Table 1:** Primers for reverse transcription PCR and qPCR assays.

Gene	Forward (5’-3’)	Reverse (5’-3’)
*β-actin*	ATTGCCGACAGGATGCAGAA	GCTGATCCACATCTGCTGGA
*IL-6*	GAGGATACCACTCCCAACAGACC	AAGTGCATCATCGTTGTTCATACA
*IL-33*	CCTGTCAACAGCAGTCTACT	TTGGCATGCAACCAGAAGTC
*TSLP*	AAATCCAGAGCCTAACCTTCAATC	CTTCATTGAGTAGCATTTATC
dfa-miR-276-3p	miRA101206 (Forward primer lot no.)	Uni-Reverse primer from C10712-1 (Reverse primer lot no.)
dfa-miR-5735-3p	miRA101208 (Forward primer lot no.)	
dfa-miR-252	miRA101207 (Forward primer lot no.)	
dfa-miR-1-3p	miRA100182 (Forward primer lot no.)	
dfa-miR-let-7b	miRA101214 (Forward primer lot no.)	
dfa-novel-miR1	miRA101209 (Forward primer lot no.)	
dfa-novel-miR2	miRA101210 (Forward primer lot no.)	
dfa-novel-miR3	miRA101211 (Forward primer lot no.)	
dfa-novel-miR4	miRA101212 (Forward primer lot no.)	
dfa-novel-miR5	miRA101213 (Forward primer lot no.)	
cel-39	miRA0000010 (Forward primer lot no.)	
U6	miRAN0002-1-200(Forward primer lot no.)	

### Enzyme-linked immunosorbent assay

BEAS-2B cells were seeded onto 24-well plates (Corning) at a density of 2×10^6^ cells/mL, and treated with 0, 1 and 10 μg/mL DFA-derived exosomes for 24 hours. The concentrations of interleukin (IL)-6, IL-33 and thymic stromal lymphopoietin (TSLP) were measured using ELISA kits (4A BIOTECH; Suzhou, China) in the cell culture supernatants following the manufacturer’s instructions.

### Cell transfection and RNA-Seq analysis

Dfa-miR-276-3p mimics (RiboBio), dfa-novel-miR2 mimics (RiboBio) and negative controls (NC mimics) (RiboBio) were transfected into BEAS-2B cells using the riboFECT CP Transfection Kit (RiboBio) following the manufacturer’s instructions, and the transfection efficiency was checked 48 hours post-transfection. Total RNA was extracted using the TRLzol Reagent (TaKaRa), and RNA purity was measured with the NanoDrop Ultramicro Spectrophotometer (Thermo Fisher Scientific). RNA integrity was evaluated with the Agilent RNA 6000 Nano Kit on the Agilent 2100 Bioanalyzer (Agilent). Total RNA was sequenced on the BGISEQ-500 High-throughput Sequencing Platform (BGI), and heat maps and volcanic maps were created based on sequencing analysis. All raw transcriptome sequencing data generated from this study have been deposited in NCBI BioProject (accession number: PRJNA1023698). Protein-protein interaction (PPI) networks were constructed using the STRING database (https://string-db.org), and a composite score of greater than 0.4 was considered as a statistically significant interaction. After unrelated genes were removed, the first 300 nodes of differentially expressed genes (DEGs) were assigned into a circle in the PPI network according to the betweenness centrality, with redder colors indicative of a higher degree of the gene. The plugin Molecular Complex Detection (MCODE) version 1.5.1 in Cytoscape (14) was used to cluster the whole network to screen the interacting key genes, with a < 0.2 limit of node score cut off. Kyoto Encyclopedia of Genes and Genomes (KEGG) pathway and Gene Ontology (GO) terms enrichment analyses were performed for each cluster. *P* < 0.05 was indicative of statistical significance.

### Statistical analysis

All measurement data were shown as mean ± standard deviation (SD). Differences of means between two groups were tested for statistical significance with Student *t* test, and a *P* value of < 0.05 was considered statistically significant. All statistical analyses were conducted using statistical software SPSS version 21.0 (SPSS., Inc.; Chicago, IL, USA).

### Ethical statement

This study was approved by the Institutional Ethical Review Committee of Jiangnan University Medical Center (approval number: 2022-Y-76). All procedures were performed following international and national laws, regulations and guidelines.

## Results

### miRNAs expression profiles in DFA samples

**Figure 1:**
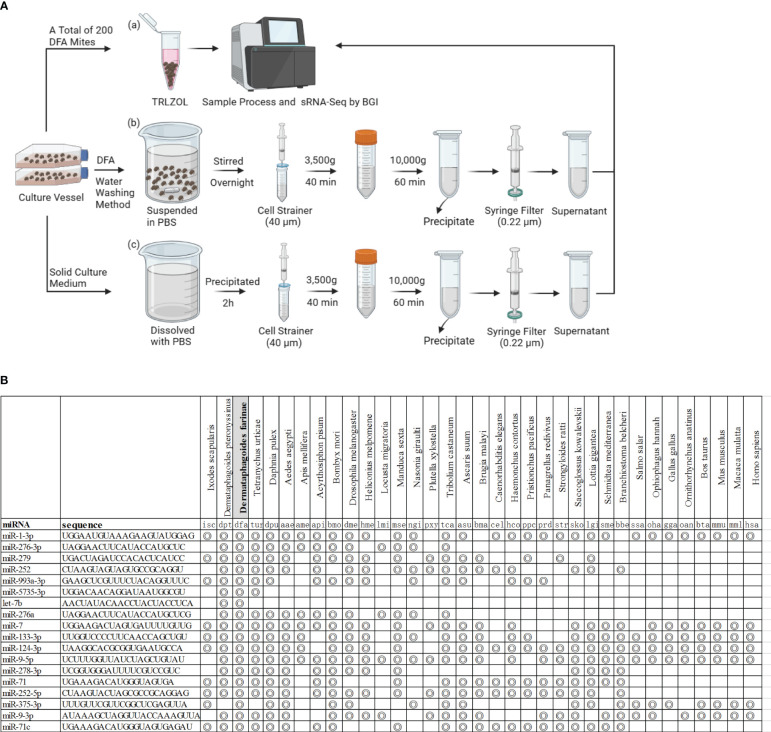
The miRNAs expression profiles in DFA-related samples. **(A)** Schematic diagram of DFA-related sample preparation for sRNA sequencing. **(B)** The figure indicates miRNAs homologues in species from different Phyla and the concentric circle-marked boxes indicate the presence of homologues in the species.

DFA mites, DFA-derived exosomes and DFA culture supernatants were prepared for sRNA-seq analysis (**Figure 1A**) and sRNA-Seq analysis identified 42 conserved miRNAs by aligning sRNAs to known miRNAs in the miRBase database (**Table 2**), and 66 novel miRNAs using the software miRDeep2 based on mature sequences and expected hairpin sequences (**Table 3**). High expression [>1,000 transcript per million (TPM)] was seen for dfa-miR-276, dfa-miR-276-3p, dfa-miR-7, dfa-miR-279, dfa-miR-1-3p, dfa-miR-5735-3p and dfa-let-7b in DFA mites and DFA-derived exosomes, which are also highly expressed in other insect species (15, 16), and dfa-novel-miR1 to dfa-novel-miR6 all presented abundant expression (>1,000 TPM) in DFA mites and DFA-derived exosomes, with comparable expression to conserved miRNAs. While the miRNAs expression was much lower in DFA culture supernatants relative to DFA mites and DFA-derived exosomes, and the expression of 7 conserved miRNAs (dfa-miR-1-3p, dfa-miR-279, dfa-miR-252, dfa-miR-5735-3p, dfa-miR-276-3p, dfa-miR-7 and dfa-miR-9a-5p) and 7 novel miRNAs (dfa-novel-miR1, 2, 3, 4, 6, 8 and 41) was relatively higher (>100 TPM) in DFA culture supernatants Then, the 18 mostly highly expressed conserved miRNAs were analyzed (**Figure 1B**), and dfa-let-7b, dfa-miR-5735-3p, dfa-miR-276-3p were relatively specific in DFA samples.

**Table 2:** Conversed miRNAs expression profile of DFA-related samples.

No.	miRNA id dfa-	Sequence (mature)	*D. farinae* UMI_TPM	Exosomes* UMI_TPM	Supernatant UMI_TPM
1	dfa-miR-1-3p	UGGAAUGUAAAGAAGUAUGGAG	4555.54	1311.46	329.04
2	dfa-miR-276	UAGGAACUUCAUACCAUGCUC	3076.32	179.06	2.32
3	dfa-miR-279	UGACUAGAUCCACACUCAUCC	2518.65	1503.29	190.44
4	dfa-miR-252	CUAAGUAGUAGUGCCGCAGGU	2097.86	627.42	694.91
5	dfa-miR-279a	UGACUAGAUCCACACUCAUCCA	1832.92	628.15	13.59
6	dfa-miR-993a-3p	GAAGCUCGUUUCUACAGGUUUC	1692.46	58.65	0.93
7	dfa-miR-5735-3p	UGGACAACAGGAUAAUGGCGU	1673.77	1105.14	484.14
8	dfa-let-7b	AACUAUACAACCUACUACCUCA	1486.09	1396.43	23.96
9	dfa-miR-276-3p	UAGGAACUUCAUACCAUGCUCG	1404.73	2889.21	675.31
10	dfa-miR-7	UGGAAGACUAGUGAUUUUGUUG	1385.23	2834.30	359.88
11	dfa-miR-133-3p	UUGGUCCCCUUCAACCAGCUGU	1375.35	974.59	34.14
12	dfa-miR-124-3p	UAAGGCACGCGGUGAAUGCCA	1158.29	334.60	8.76
13	dfa-miR-9-5p	UCUUUGGUUAUCUAGCUGUAU	952.53	93.44	82.39
14	dfa-miR-278-3p	UCGGUGGGAUUUUCGUCCGUC	893.44	53.90	12.25
15	dfa-miR-9a-5p	UCUUUGGUUAUCUAGCUGUAUG	872.96	6.57	357.70
16	dfa-miR-71	UGAAAGACAUGGGUAGUGA	789.81	244.40	24.18
17	dfa-miR-252-5p	CUAAGUACUAGCGCCGCAGGAG	770.13	232.35	29.47
18	dfa-miR-375-3p	UUUGUUCGUUCGGCUCGAGUUA	710.15	391.99	7.12
19	dfa-miR-9-3p	AUAAAGCUAGGUUACCAAAGUUA	681.56	258.92	1.15
20	dfa-miR-71c	UGAAAGACAUGGGUAGUGAGAU	617.59	660.47	2.89
21	dfa-miR-307a	UCACAACCUCCUUGAGUGAG	518.78	234.99	0.52
22	dfa-miR-184-3p	UGGACGGAGAACUGAUAAGGGC	493.06	9.90	18.01
23	dfa-miR-71a	UGAAAGACAUGGGUAGUGAGAUG	422.43	219.94	36.27
24	dfa-miR-7a	UGGAAGACUAGUGAUUUUGUUGUU	375.54	40.68	23.85
25	dfa-miR-279a	UGACUAGAUCCACACUCAU	367.60	112.91	0.96
26	dfa-miR-133-3p	UUGGUCCCCUUCAACCAGCUG	320.17	245.01	2.32
27	dfa-miR-124a-3p	UAAGGCACGCGGUGAAUGCC	284.61	106.46	2.05
28	dfa-miR-184a-3p	UGGACGGAGAACUGAUAAGGG	283.00	8.15	21.61
29	dfa-miR-7b	UGGAAGACUAGUGAUUUUGUUGU	254.19	1093.67	41.84
30	dfa-miR-9b-5p	UCUUUGGUUAUCUAGCUGUAUGA	217.30	8.60	4.34
31	dfa-miR-124b-3p	UAAGGCACGCGGUGAAUGCCAAG	195.49	4.70	0.11
32	dfa-miR-124c-3p	UAAGGCACGCGGUGAAUGCCAA	121.77	13.67	0.55
33	dfa-miR-137-3p	UUAUUGCUUGAGAAUACACG	55.01	24.25	1.28
34	dfa-miR-9c-5p	CUUUGGUUAUCUAGCUGUAUGA	50.07	8.40	0
35	dfa-miR-7c	UGGAAGACUAGUGAUUUUGUUGUUC	37.26	0.57	0
36	dfa-miR-124d-3p	UAAGGCACGCGGUGAAUGC	35.41	38.45	1.15
37	dfa-miR-210-3p	UUGUGCGUGUGACAGCGGCU	34.83	2.64	2.48
38	dfa-miR-210a-3p	CUUGUGCGUGUGACAGCGGCUAU	9.57	0.28	0.55
39	dfa-miR-307-3p	UCACAACCUCCUUGAGUGAGUGA	9.00	0.20	0.55
40	dfa-miR-210b-3p	UUGUGCGUGUGACAGCGGCUA	4.85	0.49	0
41	dfa-miR-234	UUAUUGCUUGAGAAUACA	0.12	0.12	0.22
42	dfa-miR-307b-5p	UCACUCAAGGAGGUUGUGAUG	0.09	0.04	0

^#^UMI_TPM, unique molecular identifiers_transcript per million;

*:Expression profile of miRNAs from DFA exosomes was partly published in our previous report (11).

**Table 3:** Novel miRNAs expression profile of DFA-related samples.

No.	miRNA id dfa-	Sequence (mature)	*D. farinae* UMI_TPM	Exosomes* UMI_TPM	Supernatant UMI_TPM
1	novel-miR1	UAUCACAGCCUUUUUGAUGUCU	4749.25	6535.71	1458.20
2	novel-miR2	UAUCACAGCCUAGUUAACACGAU	6806.67	5605.86	1124.98
3	novel-miR3	UGAUAUGUUUGAUAUUCUUGGUU	4081.17	4321.37	169.64
4	novel-miR4	UAUCACAGCCACUUUGAUUAGU	5188.15	4047.45	938.75
5	novel-miR5	UAUCACAGCCAGCUUUGGUGAGU	6271.48	1077.60	98.36
6	novel-miR6	AACACAUCUAGCUUGUAAGGAUU	2756.31	2441.22	802.13
7	novel-miR7	GAGAAAGGUGCCCGUCAAGUCU	0	1641.51	0
8	novel-miR8	UAAGGCCUUUAUGUUUCGUAUGA	693.98	1465.78	497.89
9	novel-miR9	GCGCGAUUGGACCCGUGCUGACGUC	0	2702.37	0
10	novel-miR10	AUAAGAUUUUGAAACGACAAGA	0	351.91	49.01
11	novel-miR11	UACGGUCCUCUUGUGUGCCUUU	595.99	312.33	84.96
12	novel-miR12	UUCGUAAUAAGUUUAACGGAC	185.71	114.01	58.73
13	novel-miR13	UUGACUAGAACUCACCUUCGUA	471.65	110.03	0
14	novel-miR14	UUGUAUACUAAAGUGAGGAUCU	4.63	45.95	0
15	novel-miR15	UAAGAUAACUUAUUACGGUUGG	190.25	35.20	5.46
16	novel-miR16	UGCGAUCAUUUUGCAUUGUUGGUU	0	22.10	0
17	novel-miR17	UUCCAACAUUUGAACAUUUUAAG	6.48	9.86	17
18	novel-miR18	UGAUUGGCUCGUGGAUGUUACAUC	0	5.39	2.62
19	novel-miR19	UGGAUUCAGAGAUGUCGUACCAGU	0	10.42	0
20	novel-miR20	UGAUUUUAUUGUUUGAAUGUCGGU	0	2.88	0
21	novel-miR21	UCGAUGAAACUAGACAAUGAUGUU	0	1.46	0
22	novel-miR22	UGUAACUCGUUAGCGCUGU	0	2.80	0
23	novel-miR23	UUACGUAUUUUUUCCCGUUCGUUU	0	1.18	0
24	novel-miR24	GGAACCACGCUCUGCUACCA	0	0.81	0
25	novel-miR25	UGGUUACCAUUCGGUCGAGGUU	0	0.57	0
26	novel-miR26	UUGUAGUCGCACCGCCACCACC	0.55	0.61	0
27	novel-miR27	AAAUCUUGUACCAAAUUGACCAUG	0	0.97	0
28	novel-miR28	UGGUAACGUUGUUCAUUGACAGG	0	0.69	0
29	novel-miR29	AGCUUACGACCAUAUCACG	154.27	163.73	65.53
30	novel-miR30	UGAAAAGUUGGAGCUGCGAGGCC	0	6.33	0
31	novel-miR31	AGGAGAUCCAUGGGUUCAAAGUGG	0	0.49	0
32	novel-miR32	UCGAUGAUGGCCAAAACGUUGCG	0	0.49	0
33	novel-miR33	UGGCAGUGUGGUUAGCUUGGUU	8318.91	0	0
34	novel-miR34	UCACCGGGUUUUCGACACCGUU	236.51	0	0
35	novel-miR35	UGUGAACGCUGCAACCAAUCAUUC	1.48	0	0
36	novel-miR36	UGGCUCGCGAGCCGCAGUUUGCC	3.21	0	0
37	novel-miR37	CUUGGCUGUUUUCAUUUUGGACAU	1.24	0	0
38	novel-miR38	UGACUGGAUAUUGAGACGAUUUUU	0.33	0	0
39	novel-miR39	UGUGGACGACGUUGUGUGAGCGAC	198.37	0	0
40	novel-miR40	UUCCGGUCAAUCGAGUUUCAGGUU	0.39	0	0
41	novel-miR41	AGGUUAGGUUAGGUUAGGUUAGG	0	0	438.51
42	novel-miR42	UGAGCAACGUUUUCGAAUUUUCAGU	0	0	1.23
43	novel-miR43	UGAUGAUGAUGAUGAUGAUGA	0	0	1.88
44	novel-miR44	GUGGUGGUGGUGGUGGUGGUGGUG	0	0	4.04
45	novel-miR45	UUUGAACAACAACAACAACAAC	0	0	3.38
46	novel-miR46	UGGCUCGAUUAUCAAUGUGGAU	0	0	1.66
47	novel-miR47	GUGUGUGUGUGUGUGUGA	0	0	3.60
48	novel-miR48	UAAUUAUUAUGUCCGACCAAAA	0	0	1.86
49	novel-miR49	GGACAGGAAAGACCGUGGC	0	0	1.01
50	novel-miR50	GGUGUUGCUAUCGUAUCUGGG	0	0	0.82
51	novel-miR51	UUUCGCUUGAGUCUUGGCGUU	0	0	0.76
52	novel-miR52	AGAUUUUUGCAUGACUUACGUUG	0	0	0.71
53	novel-miR53	UAAAUGCACAACCACAUUGUCAACA	0	0	0.68
54	novel-miR54	UGCGUGGACCAAAUUCUGUUCGG	0	0	0.60
55	novel-miR55	UAAAGGUUCCAUUGUUUGCAUUU	0	0	0.60
56	novel-miR56	UGGAAAGAGUCAAUAAAAAA	0	0	0.60
57	novel-miR57	UGUAUGGUAUUGGAUCUC	0	0	0.55
58	novel-miR58	ACCACGCUCUGCUACCAAU	0	0	0.55
59	novel-miR59	UACUGCUGAUUUGUUGUUGUUGU	0	0	0.49
60	novel-miR60	UCUCCGAUUUAAUGCAUUUGUUUU	0	0	0.46
61	novel-miR61	AGACAACGAGUGGACAGGCCAUGU	0	0	0.44
62	novel-miR62	GUGUGCGUGCGUGUAUUGA	0	0	0.44
63	novel-miR63	GUGGUCAUUGCCAUCGUCGGUGUCA	0	0	0.41
64	novel-miR64	AGGGCAUUGAUGGUCCAGUUGGU	0	0	0.38
65	novel-miR65	UACCCAAUAUCCUGUCAAUGAAC	0	0	0.35
66	novel-miR66	CAUUCCGUUUGUUGAACGACG	0	0	0.35

^#^UMI_TPM, unique molecular identifiers_transcript per million.

*:Expression profile of miRNAs from DFA-derived exosomes was partly published in our previous report (11).

### Verification of miRNA expression in DFA by qPCR assay

**Figure 2:**
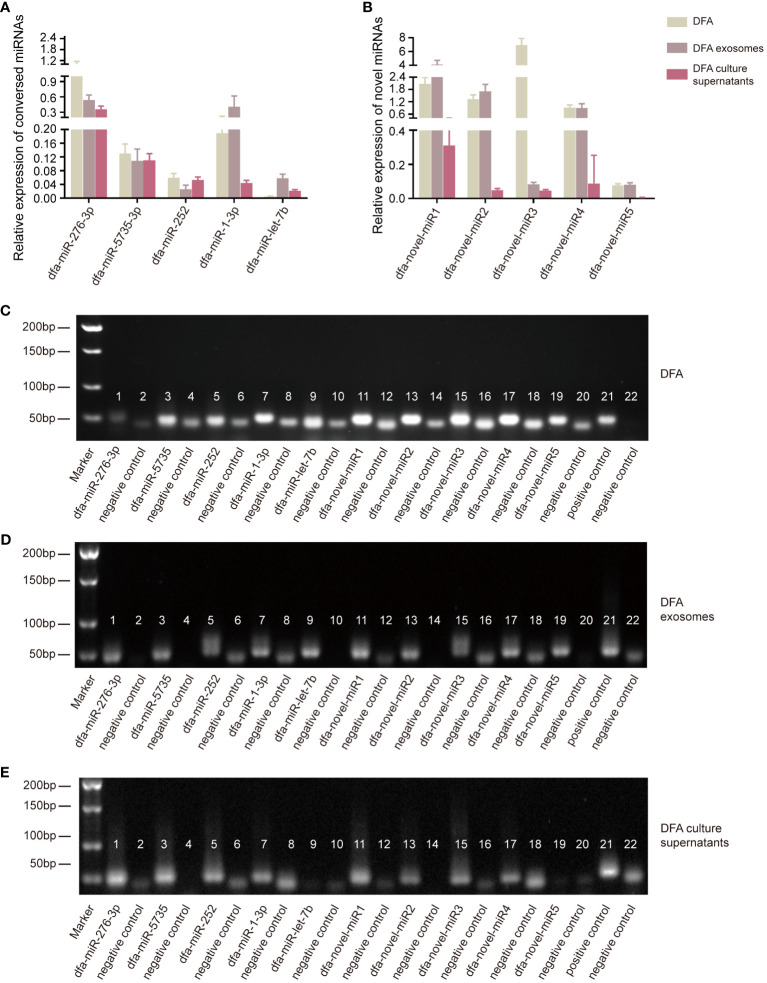
Verification of miRNAs expression in DFA by qPCR assay. **(A, B)** The poly (A)-tailing technique miRNA assay of conserved (dfa-miR-276-3p, 5735, 252, 1-3p, let-7b) and novel miRNAs (dfa-novel-miR1, 2, 3, 4, 5) in DFA mites, DFA-derived exosomes and DFA culture supernatants. **(C–E)** Electrophoresis detects the expression of conserved and novel miRNAs. Lane 1, 3, 5, 7, 9, 11, 13, 15, 17 and 19 are corresponding to dfa-miR-276-3p, -5735, -252, -1-3p, -let-7b, -novel-miR1, 2, 3, 4, 5; Lane 2, 4, 6, 8, 10, 12, 14, 16, 18 and 20 are negative controls of corresponding miRNAs and there is no template in these PCR reaction systems. Lane 21 is an external positive control cel-miR-39 of all these PCR system and Lane 22 is a negative control of Lane 21.

The presence of these conserved and novel miRNAs was verified using qPCR assay, with templates transcribed from RNA samples of DFA mites, DFA-derived exosomes and DFA culture supernatants using the poly (A)-tailing technique (11). qPCR assay detected lower expression of dfa-miR-276-3p, dfa-miR-5735-3p, dfa-miR-252, dfa-miR-1-3p, dfa-miR-let-7b and dfa-novel-miR1, 2, 3, 4 and 5 in DFA culture supernatants than in DFA mites and DFA-derived exosomes (**Figures 2A, B**), with dfa-miR-276-3p as the most highly expressed conserved miRNA in DFA samples (**Figure 2A**), and dfa-novel-miR1, 2 and 4 presenting relatively higher expression than other novel miRNAs in DFA mites and DFA-derived exosomes (**Figure 2B**).

Next, electrophoresis was performed to detect miRNAs expression (**Figures 2C–E**), and dfa-miR-1-3p presented the highest expression in DFA samples; however, dfa-miR-276-3p was more suitable to be selected for the subsequent cell transfection because of the specificity of dust mites and qPCR results (**Figure 2A**). In addition, dfa-novel-miR2 was identified as the most highly expressed novel miRNA in DFA mites, DFA-derived exosomes and DFA culture supernatants. Therefore, dfa-miR-276-3p and dfa-novel-miR2 were selected for subsequent cell transfection assays.

### Verification of HDMs miRNA in dust samples

**Figure 3:**
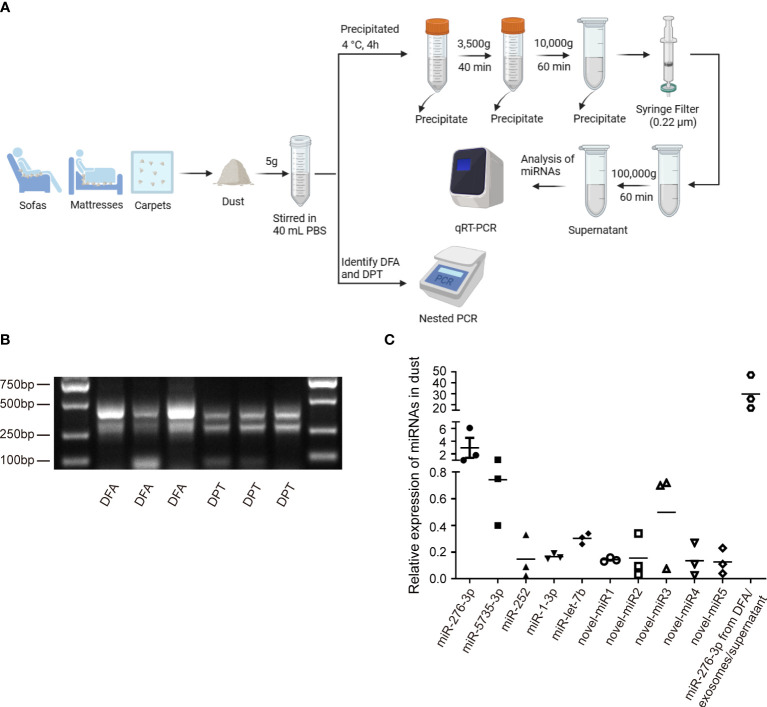
Verification miRNA expression in HDMs. **(A)** Schematic diagram of dust collection and analysis. **(B)** Nest PCR assay identifies DFA and DPT in dust samples collected from daily living environments. **(C)** qPCR assay quantifies expression of conserved (dfa-miR-276-3p, 5735, 252, 1-3p, let-7b) and novel miRNAs (dfa-novel-miR1, 2, 3, 4, 5) in HDMs from dust-related samples. Dfa-miR-276-3p from DFA, DFA-derived exosomes and DFA culture supernatants is detected as a positive control.

To verify the presence of dust mites in environments, PCR assay was performed to detect DFA and DPT in household dust samples (**Figure 3A**). Dust samples from the curtains, sofas, mattresses and carpets of inhabited houses, the vacant houses and the students’ dormitories were collected, and as expected, DFA and DPT were both detected in dust samples (**Figure 3B**). Next, qPCR assay was performed to identify highly expressed miRNAs in dust mites (**Figure 3A**), and differential expression of miR-276-3p, miR-5735-3p, miR-252, miR-1-3p, let-7b and novel-miR1, 2, 3, 4 and 5 was identified in dust samples (**Figure 3C**). Our findings confirmed the presence of miRNAs secreted by HDMs in dust samples.

### Pro-inflammatory effect of DFA-derived exosomes on BEAS-2B cells

**Figure 4:**
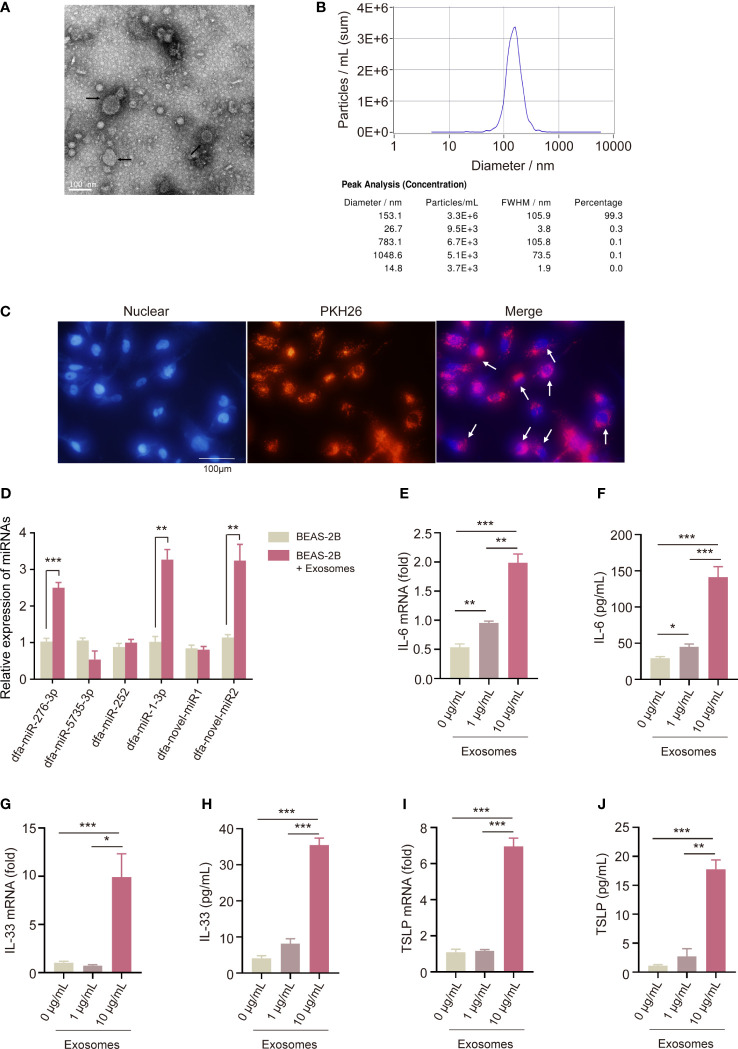
Pro-inflammatory effect of DFA-derived exosomes on BEAS-2B cells. **(A, B)** Transmission electron microscopy **(A)** and nanoparticle tracking analysis **(B)** are used to detect specific characteristics of DFA-derived exosomes. **(C)** Uptake of PKH26-labelled DFA-derived exosomes by BEAS-2B cells using immunofluorescence assay. **(D)** The poly **(A)**-tailing technique miRNA assay of conserved and novel miRNAs (dfa-miR-276-3p, 5735, 252, 1-3p, dfa-novel-miR1, 2) in DFA-derived exosomes taken up by BEAS-2B cells. **(E–J)** The levels of IL-6, IL-33 and TSLP in BEAS-2B cells stimulated by 0, 1, 10 μg/mL DFA-derived exosomes. **(E, G, I)** qPCR assay quantifies IL-6, IL-33 and TSLP mRNA expression; **(F, H, J)** ELISA assay detected IL-6, IL-33 and TSLP levels. All data are expressed as mean ± SEM from at least three independent experiments, *P < 0.05, **P < 0.01, ***P < 0.001.

Transmission electron microscopy displayed characteristic cup-shaped and lipid bilayer morphology in DFA-derived exosomes (**Figure 4A**), and NTA measured a mean diameter of 150 nm and a 10^9^/mL particle concentration of DFA-derived exosomes (**Figure 4B**).

To confirm the uptake of DFA-derived exosomes by BEAS-2B cells, we labeled DFA-derived exosomes with PKH26 dyes for 24 hours. The uptake of PKH26-labeled DFA-derived exosomes was observed by BEAS-2B cells, and fluorescent microscopy showed remarkable red fluorescence in BEAS-2B cells (**Figure 4C**).

Next, we detected the expression of conserved and novel miRNAs in BEAS-2B cells, and qPCR assay detected significant elevation of dfa-miR-276-3p, dfa-miR-1-3p and dfa-novel-miR2 expression in BEAS-2B cells loaded with DFA-derived exosomes (**Figure 4D**), indicating the entrance of DFA miRNAs into BEAS-2B cells via exosomes. To assess the role of DFA-derived exosomes in mediation of inflammatory responses of BEAS-2B cells, we compared the expression of pro-inflammatory cytokines in BEAS-2B cells stimulated by different concentrations of DFA-derived exosomes (**Figures 4E–J**). As expected, treatment with DFA-derived exosomes resulted in an increase in pro-inflammatory factors IL-6 (**Figures 4E, F**), IL-33 (**Figures 4G, H**) and TSLP (**Figures 4I, J**) in a dose-dependent manner at both translational and transcriptional levels. It is therefore considered that DFA-derived exosomes may invade bronchial epithelial cells and trigger release of pro-inflammatory cytokines, and the content of miRNAs carried by DFA-derived exosomes may be a potential cause leading to release of inflammatory factors.

### Bioinformatics analysis of BEAS-2B cells transfected with DFA miRNAs

**Figure 5:**
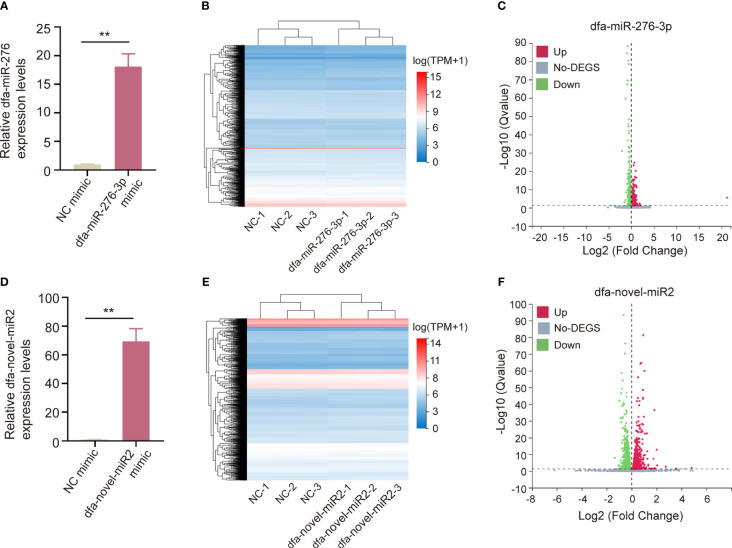
Bioinformatics analysis of BEAS-2B cells transfected with miRNAs from DFA. **(A, D)** qPCR assay checkes the transfection efficiency of dfa-miR-276-3p **(A)** and dfa-novel-miR2 **(D)** in BEAS-2B cells. **(B, E)** Heat maps of DEGs between in BEAS-2B cells transfected with dfa-miR-276-3p mimics and NC mimics **(B)** as well as between in BEAS-2B cells transfected with dfa-novel-miR2 mimics and NC mimics **(E)**. Blue represents low expression and red represents high expression. **(C, F)** Volcano plots show DEGs between in BEAS-2B cells transfected with dfa-miR-276-3p mimic **(C)** and dfa-novel-miR2 mimic **(F)** (*Q <*0.05).

According to the species specificity and expression of DFA miRNAs, we selected dfa-miR-276-3p and dfa-novel-miR2 for cell transfect into BEAS-2B cells, and qPCR assay showed high transfection efficiency of dfa-miR-276-3p (**Figure 5A**) and dfa-novel-miR2 (**Figure 5D**). Following normalization of sequencing data, heat maps were plotted (**Figures 5B, E**). In addition, qPCR assay identified 911 downregulated genes and 463 upregulated genes in BEAS-2B cells transfected with dfa-miR-276-3p mimics relative to cells transfected with NC mimics (*Q* < 0.05) (**Figure 5C**), and revealed 1,136 downregulated genes and 1,050 upregulated genes in BEAS-2B cells transfected with dfa-novel-miR2 mimics relative to cells transfected with NC mimics (*Q* < 0.05) (**Figure 5F**).

### GO terms and KEGG pathway enrichment analyses of DEGs

**Figure 6:**
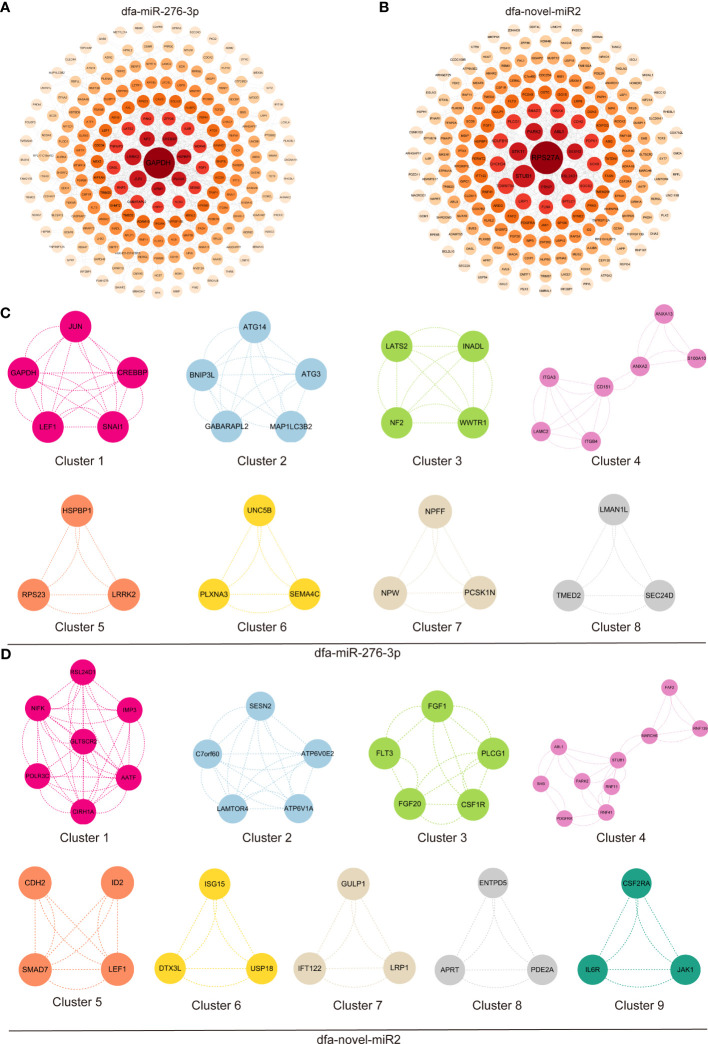
GO terms and KEGG pathway enrichment analysis of DEGs. **(A, B)** PPI networks constructed by the STRING database and optimized with the Cytoscape software, display the first 300 nodes of DEGs between in BEAS-2B cells transfected with dfa-miR-276-3p **(A)** and dfa-novel-miR2 **(B)**. **(C, D)** The first 300 nodes of DEGs between in BEAS-2B cells transfected with dfa-miR-276-3p and dfa-novel-miR2 are divided into 8 clusters **(C)** and 9 clusters **(D)** according to functions, and PPI networks are plotted for DEGs in each cluster.

The first 300 nodes of DEGs were loaded into PPI networks using the STRING database, followed by visualization with the Cytoscape software (**Figures 6A, B**). The first 300 nodes of DEGs between in dfa-miR-276-3p mimics- and NC mimics-transfected BEAS-2B cells were classified 8 clusters (**Figure 6C**), and GO terms and KEGG pathway enrichment analyses revealed that DEGs in Cluster 1 were mainly enriched with positive regulation by host of viral transcription, and those in clusters 2 and 3 were mainly enriched in cellular response to nitrogen starvation and hippo signaling pathways, (**Tables S1**, **S2**). In addition, the first 300 nodes of DEGs between in dfa-novel-miR2 mimics- and NC mimics-transfected BEAS-2B cells were classified into 9 clusters (**Figure 6D**), and GO terms and KEGG pathway enrichment analyses showed DEGs in Cluster 1 were mainly enriched in ribosomal large subunit biogenesis, and those in clusters 2 and 3 were mainly enriched in mTOR and Ras signaling pathways (**Tables S3**, **S4**). These results indicate that DFA in daily living environments secretes exosomes into bronchial epithelial cells, and then exosomes release miRNAs, which may participate in allergic inflammation of the airway epithelium via inflammatory signaling pathways.

## Discussion

**Figure 7:**
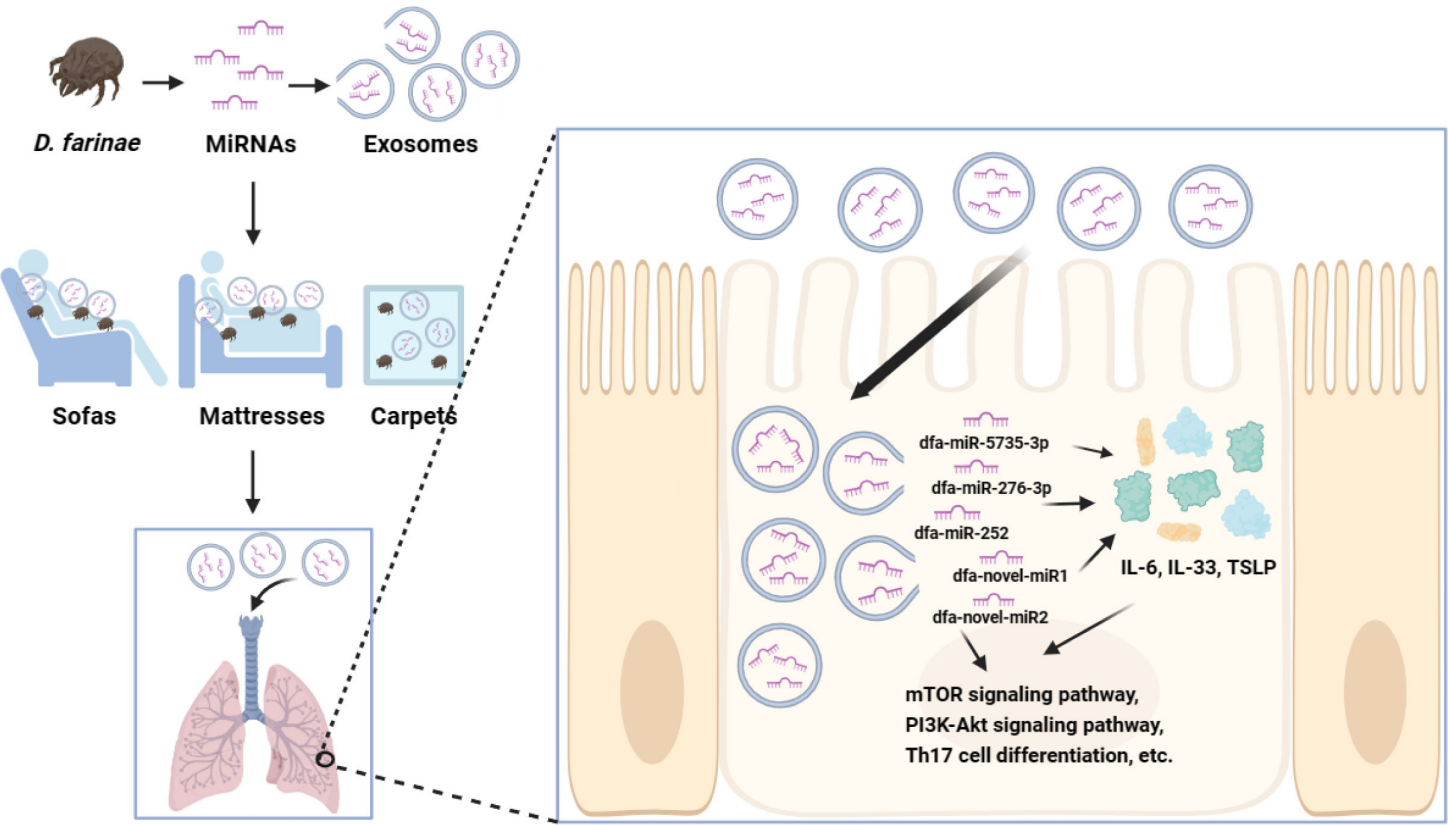
The potential mechanisms of DFA miRNAs sensitization. DFA-derived exosomes in daily living environments are inhaled into the bronchial epithelial cells, and exosomes release miRNAs, such as dfa-miR-276-3p and dfa-novel-miR2, which stimulate the expression of IL-6, IL-33, TSLP, and possibly regulate inflammatory signaling pathways, such as mTOR, PI3K-Akt and Th17 cell differentiation signaling pathways.

The sensitization mechanisms of allergens Der f1 and Der f2 have been extensively investigated (17, 18); however, the exact role of DFA miRNAs in allergic airway diseases remains unclear until now. Our previous study unraveled the miRNA expression profile of DFA-derived exosomes and preliminarily demonstrated their sensitizing effects (11). In this study, we further investigated the miRNA expression profiles of DFA mites and DFA culture supernatants, and found that DFA-derived exosomes delivered DFA-specific miRNAs into external environments. In addition, we found DFA-derived exosomes transmitted miRNAs into BEAS-2B cells and induced secretion of inflammatory factors, and RNA-Seq analysis revealed the changes in the transcriptome of BEAS-2B cells post-transfection with dfa-miR-276-3p and dfa-miR-novel2. It is therefore hypothesized that DFA miRNAs are delivered into living environments via exosomes, and uptake of DFA-derived exosomes by human bronchial epithelial cells results in cross-species regulation that may contribute to inflammation-related biological processes (**Figure 7**).

In the current study, we sequenced the miRNA expression profiles in three types of DFA-related samples, including DFA mites, DFA-derived exosomes, and DFA culture supernatants, and sRNA-Seq analysis identified 42 conserved miRNAs and 66 novel miRNAs, including 42 conserved miRNAs and 23 novel miRNAs in DFA mites, and 42 conserved miRNAs and 32 novel miRNAs in DFA-derived exosomes, with comparable miRNAs expression profiles between DFA mites and DFA-derived exosomes, suggesting that miRNAs derived from DFA mites are packaged and carried by exosomes and possibly secreted to living environments. In addition, we identified 38 conserved miRNAs and 40 novel miRNAs in DFA culture supernatants, and the highly expressed miRNAs were comparable among these three types of DFA-related samples; however, the miRNAs expression was lower in DFA culture supernatants than in DFA mites and DFA-derived exosomes. It is therefore hypothesized that the miRNAs in the solid culture medium were actively secreted by DFA in the form of exosomes, which may explain the lower miRNAs expression in DFA culture supernatants than in DFA-derived exosomes, and the diverse expression of novel miRNAs in external environments may be attributed to complex composition of the solid culture medium, where the source of miRNAs is not limited to DFA.

HDMs allergy is caused by human exposure to dust mite allergens present in external living environments (19). The presence of dust mite-derived miRNAs in external environments is a prerequisite for studying their sensitizing effects. Therefore, we selected dust samples collected from inhabited houses, vacant houses, and students’ dormitories to detect miRNAs from dust mites. First, PCR assay was used to identify the presence of DPT and DFA in collected indoor dusts. Then, qPCR assay detected relatively higher expression levels of miR-276-3p, miR-5735-3p, novel-miR2 and 3. qPCR assay results were consistent with the comparison of high-abundance expressed miRNAs in DFA-related samples, which further confirmed the presence of dust mites-derived miRNAs in daily living environments and suggested a high possibility of their existence in the form of dust mites-derived exosomes.

Next, we created a heap map of 18 most highly expressed conserved miRNAs in DFA-related samples, and these miRNAs had high homology with the Arthropoda clade and shared homology with the Chordata clade (20). Previous studies have shown that the expression level of miR-1 in peripheral blood of children suffering from acute-stage asthma positively correlated with interferon (IFN)-γ expression and negatively correlated with disease severity (21). The metabolic balance of miR-276 determined the reproductive cycle of mosquitoes and the development of *Plasmodium falciparum* (22), and miR-276a played an important role in fragile X mental retardation protein (FMRP)-mediated spatial filling dendritic morphogenesis in *Drosophila melanogaster* (2). However, our study reported for the first time that miR-276-3p was highly expressed in the *in-vitro* model of allergic disease. In addition, miR-5735-3p has been predicted to be specific in DPT and significantly influence the type I IFN signaling pathway through GO enrichment analysis of DEGs (23). Nevertheless, the role of DFA miRNAs in dust mite sensitization remains unclear until known.

It has been reported that the microvesicles secreted by intestinal epithelial cells deliver exogenous plant miRNAs to other organs and regulate the expression of mammalian target genes (24). The novel miRNA-33 from egg-derived exosomes of *Schistosoma japonicum* was found to promote liver fibrosis in hosts in a cross-species manner (25). It was reported that miR-CM1 derived from *Phellinus linteus*, a classical medicinal fungus, inhibited the expression level of Mical2 in human skin cells and inhibited ultraviolet induced skin aging in a cross-kingdom manner (26). In addition, host Arabidopsis cells inhibited *Botrytis cinerea* virulence genes by delivering miRNAs through exosomes, and inhibited fungal infection through a transboundary nucleic acid delivery mechanism (27). These results suggest that exosomes effectively transport miRNAs to recipient cells, and then present biological functions (28).

In the present study, sRNA-Seq analysis identified high expression of several conserved and novel miRNAs in DFA mites, DFA-derived exosomes and DFA culture supernatants, including dfa-miR-1-3p, dfa-miR-279, dfa-miR-252, dfa-miR-5735-3p, dfa-miR-276-3p, dfa-miR-7, and dfa-novel-miR1, 2, 3, 4, 6, 8. We hypothesized that exosomes secreted by DFA carry a portion of DFA miRNAs and then release miRNAs into DFA culture supernatants. Then, immunofluorescent staining showed uptake of DFA-derived exosomes by BEAS-2B cells, and qPCR assay further demonstrated the release of DFA miRNAs from exosomes into BEAS-2B cells. Peripheral blood IL-6 has recently been identified as a potential biomarker for adult asthma (29), and IL-33 is a susceptibility gene for childhood asthma (30), while TSLP is associated with initiation and persistence of inflammatory pathways in asthma (31). As expected, qPCR assay showed that the expression of inflammatory factors significantly increased with the concentration of DFA-derived exosomes. It is therefore considered that DFA-derived exosomes cause inflammatory responses following entrance into BEAS-2B cells and may exert pro-inflammatory effects through releasing miRNAs. Previous studies have shown that dust mites cause allergic diseases through allergens (32, 33). In this study, we found that DFA-derived exosomes promoted the secretion of inflammatory factors and exhibited high expression of exosome-delivered miRNAs in BEAS-2B cells. It is therefore hypothesized that DFA miRNAs may be the main cause leading to release of cellular inflammatory factors.

In the current study, our RNA-Seq analysis identified 1,374 DEGs in between dfa-miR-276-3p mimics- and NC mimics-transfected BEAS-2B cells (*Q* < 0.05), including 463 upregulated genes and 911 downregulated genes, and 2,186 DEGs in between dfa-novel-miR2 mimics- and NC mimics-transfected BEAS-2B cells (*Q* < 0.05), including 1,050 upregulated genes and 1,136 downregulated genes, demonstrating that DFA miRNAs regulate gene expression in bronchial epithelial cells. MCODE analysis demonstrated that the first 300 nodes of DEGs between in dfa-miR-276-3p mimics- and NC mimics-transfected BEAS-2B cells were classified into 8 clusters, and those between dfa-novel-miR2 mimics- and NC mimics-transfected BEAS-2B cells were classified into 9 clusters, confirming their different roles in the development of airway inflammation. KEGG pathway and GO terms enrichment analysis revealed that these DEGs in clusters were significantly enriched in biological processes of regulation of apoptotic process, Th17 cell differentiation and JAK-STAT and Ras signaling pathways. Targeting PI3K/AKT/mTOR signaling has been found to attenuate asthma pathology and play a significant role in airways protection (34). In this study, DEGs in Cluster 4 between in BEAS-2B cells transfected with dfa-miR-276-3p mimics and NC mimics and Cluster 3 between in BEAS-2B cells transfected with dfa-novel-miR2 mimics and NC mimics were found to regulate the PI3K/AKT signaling pathway. TGF-β has been reported to be involved in the airway inflammation and fibrotic tissue remodeling in asthma (35). In the present study, DEGs in Cluster 5 between in BEAS-2B cells transfected with dfa-novel-miR2 mimics and NC mimics were associated with TGF-β signaling pathway, and DEGs in Cluster 9 between in BEAS-2B cells transfected with dfa-novel-miR2 mimics and NC mimics were found to regulate IL-6-mediated signaling pathways, as evidenced by a significant increase of IL-6 expression following co-culture of DFA-derived exosomes and BEAS-2B cells.

Due to the presence of external RNases and other substances, *in vitro* miRNAs are easily degraded (36). In this study, we found that DFA-derived exosomes encapsulated miRNAs and released them into external environments, which may improve the miRNAs stability (11). Avoiding contact with mite allergens is crucial for treatment of dust mite sensitization (37). Currently, use of physical barriers, hot air (110°C) to kill dust mites and tannic acid for denaturing mite allergens are attempted to reduce exposure to allergens (19, 38, 39). Nevertheless, loss of bioactive miRNAs requires ultra-high-temperatures (135°C) (40). In addition, tannic acid was used as a material for constructing implants with miRNA functions (41). Therefore, miRNAs from dust mites may be novel targets for management of mite sensitization, in addition to interventions against mite allergens (42).

Taken together, the results of the present study demonstrate that exosomes are one of the pathways for DFA to secrete miRNAs to sensitize target cells. However, the role of miRNAs and DFA-derived exosomes in allergic diseases remains to be investigated. In our future studies, we will also focus on highly abundant and specifically expressed miRNAs, such as dfa-miR-276-3p, to investigate their roles in HDMs sensitizing effects and the underlying mechanisms of sensitization. Overall, our study provides new insights into allergy management and improves the quality of life among individuals with dust mite allergies.

## Data availability statement

The sRNA-seq data presented in the study are deposited in the NCBI BioProject repository, accession number PRJNA1023682, and the RNA-seq data presented in the study are deposited in the NCBI BioProject repository, accession number PRJNA1023698.

## Ethics statement

This study was approved by the Institutional Ethical Review Committee of Jiangnan University Medical Center (approval number: 2022-Y-76).

## Author contributions

KH: Investigation, Methodology, Visualization, Writing – original draft. TY: Investigation, Resources, Supervision. JY: Data curation, Formal analysis, Software. XZ: Methodology, Resources, Visualization. SJ: Resources, Software. SX: Software; JL: Data curation, Formal analysis. ZX: Methodology, Resources. WW: Project administration, Supervision, Visualization, Writing – review & editing. SH: Conceptualization, Data curation, Funding acquisition, Methodology, Supervision, Writing – original draft.
