# Supplementary material for: Dermatophagoides farinae microRNAs released to external environments via exosomes regulate inflammation-related gene expression in human bronchial epithelial cells
Source: Front Immunol. 2023 Dec 1;14:1303265. doi: 10.3389/fimmu.2023.1303265 (PMC10722260; doi:10.3389/fimmu.2023.1303265)
Supplement: Supplementary file 1 [file DataSheet_1.docx]

***Dermatophagoides farinae* microRNAs released to external environments via exosomes regulate inflammation-related gene expression in human bronchial epithelial cells**

**Supplementary document**

**Figure S1** Length distribution of sRNA: the x-axis indicates the length of sRNA and the y-axis shows the count of sRNA with specific length. The following three figures display the length distribution of sRNA from DFA mites, DFA-derived exosomes and DFA culture supernatant.


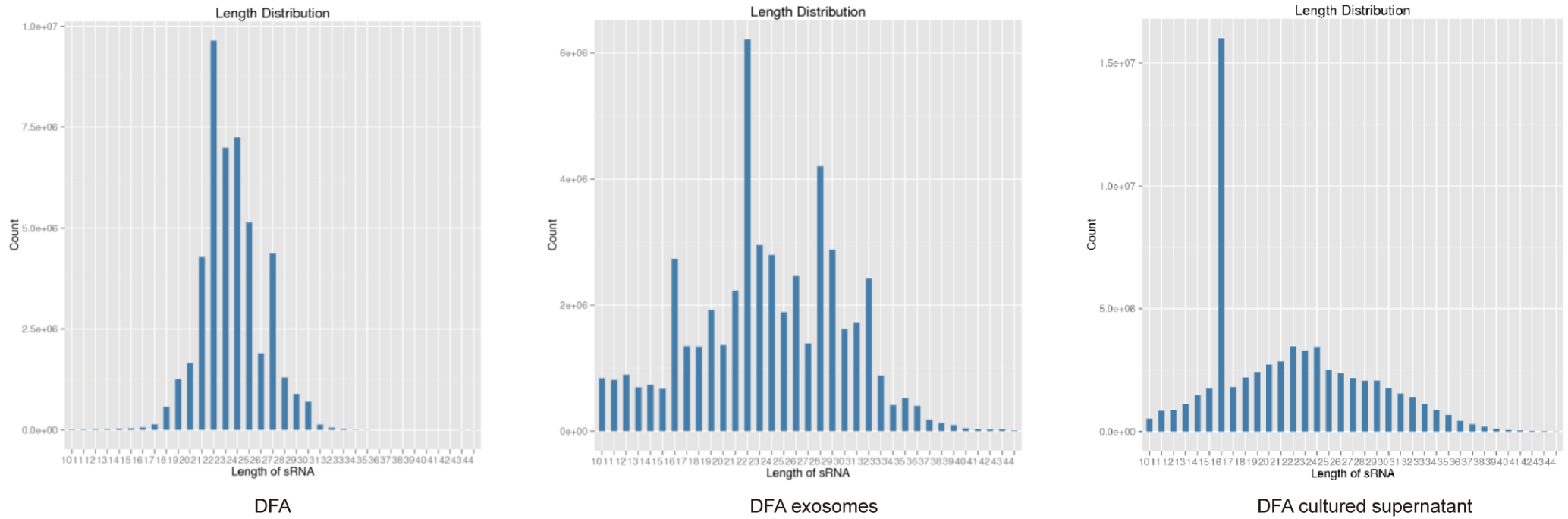


**Table S1** GO terms enrichment of analysis Dfa-miR-276 clusters

| **Cluster** | **DEGs** | **GO** | **Description** | **Strength** | **False discovery rate** |
| --- | --- | --- | --- | --- | --- |
| Cluster1 | GAPDH, CREBBP, LEF1, JUN, SNAI1 | GO:0010629 | Negative regulation of gene expression | 0.99 | 0.0146 |
|  |  | GO:0042981 | Regulation of apoptotic process | 1.1 | 0.0146 |
|  |  | GO:0043923 | Positive regulation by host of viral transcription | 2.66 | 0.0146 |
|  |  | GO:0051240 | Positive regulation of multicellular organismal process | 1.04 | 0.0146 |
|  |  | GO:0051702 | Interaction with symbiont | 2.1 | 0.0146 |
|  |  | GO:0051817 | Modulation of process of other organism involved in symbiotic interaction | 2.08 | 0.0146 |
|  |  | GO:2000113 | Negative regulation of cellular macromolecule biosynthetic process | 1.12 | 0.0146 |
|  |  | GO:0043903 | Regulation of symbiotic process | 1.71 | 0.015 |
|  |  | GO:0044403 | Symbiotic process | 1.26 | 0.016 |
|  |  | GO:0000122 | Negative regulation of transcription by rna polymerase ii | 1.24 | 0.0171 |
| Cluster2 | GABARAPL2, ATG14, ATG3, BNIP3L, MAP1LC3B2 | GO:0007005 | Mitochondrion organization | 1.64 | 0.0000146 |
|  |  | GO:0044248 | Cellular catabolic process | 1.05 | 0.0047 |
|  |  | GO:0000422 | Autophagy of mitochondrion | 2.59 | 0.00000142 |
|  |  | GO:0000045 | Autophagosome assembly | 2.42 | 0.00000208 |
|  |  | GO:0097352 | Autophagosome maturation | 2.59 | 0.0000622 |
|  |  | GO:0006995 | Cellular response to nitrogen starvation | 2.85 | 0.004 |
|  |  | GO:0009267 | Cellular response to starvation | 1.88 | 0.0043 |
|  |  | GO:0008637 | Apoptotic mitochondrial changes | 2.14 | 0.0476 |
| Cluster4 | ITGA3, ITGB4, LAMC2, ANXA2, CD151, ANXA13, S100A10 | GO:0007155 | Cell adhesion | 1.18 | 0.0099 |
|  |  | GO:0031581 | Hemidesmosome assembly | 2.84 | 0.00016 |
|  |  | GO:0030198 | Extracellular matrix organization | 1.52 | 0.0099 |
|  |  | GO:0001765 | Membrane raft assembly | 2.75 | 0.0116 |
|  |  | GO:0034330 | Cell junction organization | 1.36 | 0.0193 |
|  |  | GO:0048333 | Mesodermal cell differentiation | 2.33 | 0.0482 |
| Cluster6 | SEMA4C, UNC5B, PLXNA3 | GO:0007411 | Axon guidance | 1.85 | 0.0364 |
|  |  | GO:0048843 | Negative regulation of axon extension involved in axon guidance | 2.72 | 0.0364 |
|  |  | GO:0050919 | Negative chemotaxis | 2.47 | 0.0364 |
|  |  | GO:0071526 | Semaphorin-plexin signaling pathway | 2.55 | 0.0364 |
| Cluster7 | PCSK1N, NPFF, NPW | GO:0007218 | Neuropeptide signaling pathway | 2.25 | 0.0025 |
| Cluster8 | TMED2, SEC24D, LMAN1L | GO:0006888 | Endoplasmic reticulum to golgi vesicle-mediated transport | 2 | 0.0131 |
|  |  | GO:0035459 | Vesicle cargo loading | 2.72 | 0.0353 |

^#^ Strength: Log10(observed / expected). This measure describes how large the enrichment effect is. It’s the ratio between i) the number of proteins in the network that are annotated with a term and ii) the number of proteins that we expect to be annotated with this term in a random network of the same size.

^#^ False discovery rate: This measure describes how significant the enrichment is. Shown are p-values corrected for multiple testing within each category using the Benjamini–Hochberg procedure.

**Table S2**  KEGG pathway enrichment analysis of dfa-miR-276 clusters

| **Cluster** | **DEGs** | **KEGG pathways** | **Description** | **Strength** | **False discovery rate** |
| --- | --- | --- | --- | --- | --- |
| Cluster 1 | GAPDH, CREBBP, LEF1, JUN, SNAI1 | hsa04520 | Adherens junction | 2.24 | 0.00015 |
|  |  | hsa04310 | Wnt signaling pathway | 1.88 | 0.00084 |
|  |  | hsa05167 | Kaposi sarcoma-associated herpesvirus infection | 1.8 | 0.00099 |
|  |  | hsa05132 | Salmonella infection | 1.75 | 0.001 |
|  |  | hsa05211 | Renal cell carcinoma | 2.07 | 0.0079 |
|  |  | hsa05200 | Pathways in cancer | 1.36 | 0.01 |
|  |  | hsa05210 | Colorectal cancer | 1.98 | 0.01 |
|  |  | hsa04066 | HIF-1 signaling pathway | 1.87 | 0.0101 |
|  |  | hsa04916 | Melanogenesis | 1.92 | 0.0101 |
|  |  | hsa05215 | Prostate cancer | 1.91 | 0.0101 |
| Cluster 2 | GABARAPL2, ATG14, ATG3,  BNIP3L, MAP1LC3B2 | hsa04140 | Autophagy - animal | 1.96 | 0.001 |
|  |  | hsa05167 | Kaposi sarcoma-associated herpesvirus infection | 1.8 | 0.0015 |
|  |  | hsa04136 | Autophagy - other | 2.43 | 0.0027 |
|  |  | hsa04137 | Mitophagy - animal | 2.09 | 0.0091 |
| Cluster 3 | NF2, INADL, LATS2, WWTR1 | hsa04390 | Hippo signaling pathway | 2.11 | 0.00000134 |
|  |  | hsa04392 | Hippo signaling pathway - multiple species | 2.74 | 0.00000218 |
|  |  | hsa04530 | Tight junction | 1.8 | 0.0431 |
| Cluster 4 | ITGA3, ITGB4, LAMC2, ANXA2, CD151, ANXA13, S100A10 | hsa04512 | ECM-receptor interaction | 1.98 | 0.0011 |
|  |  | hsa04510 | Focal adhesion | 1.63 | 0.0061 |
|  |  | hsa04151 | PI3K-Akt signaling pathway | 1.38 | 0.0174 |
|  |  | hsa05165 | Human papillomavirus infection | 1.41 | 0.0174 |
|  |  | hsa05412 | Arrhythmogenic right ventricular cardiomyopathy | 1.87 | 0.0218 |
|  |  | hsa05222 | Small cell lung cancer | 1.78 | 0.0248 |
|  |  | hsa05410 | Hypertrophic cardiomyopathy | 1.8 | 0.0248 |
|  |  | hsa05414 | Dilated cardiomyopathy | 1.77 | 0.0248 |
| Cluster 6 | SEMA4C, UNC5B, PLXNA3 | hsa04360 | Axon guidance | 2.04 | 0.00026 |

**Table S3** GO terms enrichment analysis of dfa-novel-miR2 clusters GO analysis

| **Cluster** | **DEGs** | **GO** | **Description** | **Strength** | **False discovery rate** |
| --- | --- | --- | --- | --- | --- |
| Cluster 1 | GLTSCR2, RSL24D1, NIFK, IMP3,  CIRH1A, POLR3C, AATF | GO:0042254 | Ribosome biogenesis | 1.76 | 0.00000105 |
|  |  | GO:0006364 | rRNA processing | 1.72 | 0.0021 |
|  |  | GO:0042273 | Ribosomal large subunit biogenesis | 2.07 | 0.0048 |
|  |  | GO:0010467 | Gene expression | 0.91 | 0.0159 |
| Cluster 2 | SESN2, LAMTOR4, C7orf60, ATP6V1A  ATP6V0E2 | GO:0016241 | Regulation of macroautophagy | 1.95 | 0.00042 |
|  |  | GO:0032006 | Regulation of tor signaling | 2.08 | 0.0057 |
|  |  | GO:0071417 | Cellular response to organonitrogen compound | 1.42 | 0.0132 |
|  |  | GO:1904262 | Negative regulation of torc1 signaling | 2.66 | 0.0191 |
|  |  | GO:0032868 | Response to insulin | 1.71 | 0.0304 |
|  |  | GO:0090383 | Phagosome acidification | 2.45 | 0.0323 |
|  |  | GO:0033572 | Transferrin transport | 2.34 | 0.0427 |
|  |  | GO:0034198 | Cellular response to amino acid starvation | 2.24 | 0.0427 |
|  |  | GO:1901701 | Cellular response to oxygen-containing compound | 1.17 | 0.0427 |
| Cluster 3 | FGF20, FLT3, PLCG1, CSF1R, FGF1 | GO:0007169 | Transmembrane receptor protein tyrosine kinase signaling pathway | 1.58 | 0.00017 |
|  |  | GO:0008284 | Positive regulation of cell population proliferation | 1.33 | 0.00099 |
|  |  | GO:0071363 | Cellular response to growth factor stimulus | 1.5 | 0.0065 |
|  |  | GO:0043410 | Positive regulation of mapk cascade | 1.46 | 0.0066 |
|  |  | GO:0035556 | Intracellular signal transduction | 1.06 | 0.0083 |
|  |  | GO:0006796 | Phosphate-containing compound metabolic process | 0.97 | 0.0187 |
|  |  | GO:0048584 | Positive regulation of response to stimulus | 0.94 | 0.022 |
|  |  | GO:0006468 | Protein phosphorylation | 1.22 | 0.024 |
|  |  | GO:0071310 | Cellular response to organic substance | 0.92 | 0.024 |
|  |  | GO:0071902 | Positive regulation of protein serine/threonine kinase activity | 1.53 | 0.0377 |
| Cluster 4 | STUB1, RNF11, FAF2, MARCH6, RNF139, RNF41, PARK2, ABL1, PDGFRA, SHB | GO:0036503 | ERAD pathway | 2.05 | 0.0000069 |
|  |  | GO:0051603 | Proteolysis involved in cellular protein catabolic process | 1.36 | 0.0000189 |
|  |  | GO:0010498 | Proteasomal protein catabolic process | 1.53 | 0.0000202 |
|  |  | GO:0051865 | Protein autoubiquitination | 2.05 | 0.0000831 |
|  |  | GO:0010243 | Response to organonitrogen compound | 1.14 | 0.00014 |
|  |  | GO:0044248 | Cellular catabolic process | 0.95 | 0.00018 |
|  |  | GO:0044267 | Cellular protein metabolic process | 0.68 | 0.0018 |
|  |  | GO:0006511 | Ubiquitin-dependent protein catabolic process | 1.26 | 0.0024 |
|  |  | GO:0033554 | Cellular response to stress | 0.9 | 0.0024 |
|  |  | GO:0045732 | Positive regulation of protein catabolic process | 1.51 | 0.0024 |
| Cluster 5 | ID2, SMAD7, LEF1, CDH2 | GO:0035239 | Tube morphogenesis | 1.47 | 0.0165 |
|  |  | GO:0009887 | Animal organ morphogenesis | 1.31 | 0.0183 |
|  |  | GO:0010001 | Glial cell differentiation | 1.92 | 0.0183 |
|  |  | GO:0021537 | Telencephalon development | 1.76 | 0.0183 |
|  |  | GO:0045597 | Positive regulation of cell differentiation | 1.29 | 0.0183 |
|  |  | GO:0045619 | Regulation of lymphocyte differentiation | 1.92 | 0.0183 |
|  |  | GO:0060019 | Radial glial cell differentiation | 2.91 | 0.0183 |
|  |  | GO:0060429 | Epithelium development | 1.25 | 0.0183 |
|  |  | GO:0072359 | Circulatory system development | 1.35 | 0.0183 |
|  |  | GO:0060412 | Ventricular septum morphogenesis | 2.37 | 0.0271 |
| Cluster 9 | JAK1, IL6R, CSF2RA | GO:0070102 | IL-6-mediated signaling pathway | 2.91 | 0.0308 |

**Table S4** KEGG pathway enrichment analysis of dfa-novel-miR2 clusters KEGG analysis

| **Cluster** | **DEGs** | **KEGG pathways** | **Description** | **Strength** | **False discovery rate** |
| --- | --- | --- | --- | --- | --- |
| Cluster 2 | SESN2, LAMTOR4, C7orf60, ATP6V1A, ATP6V0E2 | hsa04150 | mTOR signaling pathway | 1.89 | 0.0016 |
| Cluster 3 | FGF20, FLT3, PLCG1, CSF1R, FGF1 | hsa04014 | Ras signaling pathway | 1.94 | 7.37E-08 |
|  |  | hsa05200 | Pathways in cancer | 1.58 | 0.00000223 |
|  |  | hsa04015 | Rap1 signaling pathway | 1.89 | 0.00000662 |
|  |  | hsa04010 | MAPK signaling pathway | 1.74 | 0.0000202 |
|  |  | hsa04151 | PI3K-Akt signaling pathway | 1.65 | 0.0000349 |
|  |  | hsa05221 | Acute myeloid leukemia | 2.07 | 0.0066 |
|  |  | hsa05218 | Melanoma | 2.04 | 0.0067 |
|  |  | hsa04640 | Hematopoietic cell lineage | 1.93 | 0.0093 |
|  |  | hsa05224 | Breast cancer | 1.73 | 0.0203 |
|  |  | hsa05226 | Gastric cancer | 1.74 | 0.0203 |
| Cluster 4 | STUB1, RNF11, FAF2, MARCH6, RNF139, RNF41, PARK2, ABL1, PDGFRA, SHB | hsa04141 | Protein processing in endoplasmic reticulum | 1.55 | 0.0239 |
| Cluster 5 | ID2, SMAD7, LEF1, CDH2 | hsa04390 | Hippo signaling pathway | 1.98 | 0.00066 |
|  |  | hsa04350 | TGF-beta signaling pathway | 2.03 | 0.0157 |
|  |  | hsa05412 | Arrhythmogenic right ventricular cardiomyopathy | 2.11 | 0.0157 |
| Cluster 8 | PDE2A, ENTPD5, APRT | hsa00230 | Purine metabolism | 2.19 | 0.0000963 |
| Cluster 9 | JAK1, IL6R, CSF2RA | hsa01521 | EGFR tyrosine kinase inhibitor resistance | 2.22 | 0.0166 |
|  |  | hsa04659 | Th17 cell differentiation | 2.11 | 0.0166 |
|  |  | hsa04630 | JAK-STAT signaling pathway | 1.91 | 0.0228 |
|  |  | hsa05163 | Human cytomegalovirus infection | 1.78 | 0.0315 |
